# 8.H. Workshop: Health equity and Chronic diseases: Public health and Primary care roles

**DOI:** 10.1093/eurpub/ckac129.501

**Published:** 2022-10-25

**Authors:** Organised by: Western University (Canada), Chair persons: Saverio Stranges (EUPHA-CHR), Tatjana Makovski (France)

## Abstract

Publicly funded health systems are based on the principle of universal access to care. However, significant gaps still exist between services needed and services received (i.e. unmet need for healthcare), especially in chronic disease management and prevention, particularly affecting marginalized groups in the society. Public Health and Primary Care should be working in integrated systems, as they are crucial in addressing health needs in the community and population at large. Several international organizations and leading scholars in the field have been advocating for the integration or primary care and public health to improve population health as well as to tackle complex issues such as multimorbidity. However, these two sectors are still working in silos across several countries, with a significant negative impact in terms of efficiency of the services provided and health care costs. The aim of this workshop will be to provide a global perspective on the issue of health equity in chronic disease management and prevention, with focus on marginalized population subgroups, such as elderly, immigrants and people from low socioeconomic status. We will discuss as well the determinants of unmet health needs for access to primary care in middle-aged and older adults, and examine the reasons for unmet need. We will further discuss about the need and strategies for enhanced integration between public health and primary care in chronic disease management and prevention.

**Key messages:**

• Public health systems are based on the principle of universal access to care. However, significant gaps still exist between services needed and services received (i.e. unmet need for healthcare).

• Public Health and Primary Care should be working in integrated systems to tackle complex issues such as multimorbidity.

